# Usefulness of second-look ultrasonography using anatomical breast structures as indicators for magnetic resonance imaging-detected breast abnormalities

**DOI:** 10.1007/s12282-019-01003-z

**Published:** 2019-08-12

**Authors:** Ayumi Izumori, Yumi Kokubu, Kazuko Sato, Naoya Gomi, Hidetomo Morizono, Takehiko Sakai, Rie Horii, Futoshi Akiyama, Takuji Iwase, Shinji Ohno

**Affiliations:** 1 Department of Breast Surgery, Takamatsu Heiwa Hospital, Takamatsu, Japan; 2 Department of Diagnostic Imaging, The Cancer Institute Hospital of the Japanese Foundation for Cancer Research, Tokyo, Japan; 3 Department of Ultrasound, The Cancer Institute Hospital of the Japanese Foundation for Cancer Research, Tokyo, Japan; 4 Department of Cytology, The Cancer Institute Hospital of the Japanese Foundation for Cancer Research, Tokyo, Japan; 5 Breast Oncology Center, The Cancer Institute Hospital of the Japanese Foundation for Cancer Research, Tokyo, Japan; 6 Department of Pathology, The Cancer Institute Hospital of the Japanese Foundation for Cancer Research, Tokyo, Japan; 7 Department of Pathology, The Cancer Institute, Japanese Foundation for Cancer Research, Tokyo, Japan

**Keywords:** MRI, Ultrasound, Anatomical landmark, Fine-needle aspiration cytology

## Abstract

**Background:**

Second-look ultrasonography (US) is commonly performed for breast lesions detected using magnetic resonance imaging (MRI), but the identification rate of these lesions remains low. We investigated if US methods using anatomical breast structures can improve the lesion identification rate of MR-detected lesions and evaluated the diagnostic performance of fine-needle aspiration cytology (FNAC) of the second-look US using the above-mentioned method.

**Methods:**

We retrospectively assessed 235 breast lesions (hereinafter, “targets”) subjected to second-look US following MRI between January 2013 and September 2015. US was employed using the conventional methods, and this assessment measured the positional relationships of lesions with regard to surrounding anatomical breast structures (glandular pattern, Cooper’s ligaments, adipose morphology, and vascular routes). Associations were assessed among the following variables: the MRI findings, target size, identification rate, and main US indicators that led to identifying the target; FNAC results and MRI findings; MRI findings and histopathological findings; and FNAC results and histopathological findings. Moreover, the sensitivity and specificity of FNAC were determined.

**Results:**

The identification rate was 99%. The main US indicators leading to identification were a glandular pattern (28–30% of lesions) and other breast structures (~ 25% of lesions). FNAC was performed for 232 targets with the following results: sensitivity of 85.7%, specificity of 91.6%, PPV of 94.1%, NPV of 92.9%, false-negative rate of 14.3%, false-positive rate of 2.1%, and accuracy of 89.7%.

**Conclusions:**

Second-look US using anatomical breast structures as indicators and US-guided FNAC are useful for refining the diagnosis of suspicious breast lesions detected using MRI.

## Introduction

In recent years, magnetic resonance imaging (MRI) has become a useful tool for preoperative diagnosis of the intraductal spread of breast cancer and for screening high-risk breast cancer patients [1–3]. MRI is capable of detecting minute lesions not found by mammography (MMG) or ultrasonography (US) [3–6]. MRI-guided biopsy system is an ideal examination for lesions identified using MRI; however, it is not universally available owing to the expensive equipment required and highly invasive nature of procedure.

Several reports have described a method using second-look US to assess lesions detected by MRI [7–10]. In these reports, US lesions were identified based on their location, shape, and size in MRI, but the identification rate was low at 23–71% [9–13] owing to reasons such as different body examination positions during MRI and US scanning [11, 12]. In contrast, in the present study, we used second-look US to identify lesions based on their location, shape, and size as well as with regard to anatomical breast structures such as the glandular pattern, Cooper’s ligaments, adipose morphology, and vascular routes.

Although lesions identified by second-look US are diagnosed using core needle biopsy (CNB) or vacuum-assisted core needle biopsy (VAB) [6, 9], fine-needle aspiration cytology (FNAC), which is less invasive than CNB or VAB, has been used to diagnose such types of tiny lesions.

Here we investigated if US methods using anatomical breast structures can improve the lesion identification rate of MR-detected lesions and evaluated the diagnostic performance of fine-needle aspiration cytology (FNAC) of the second-look US using the above-mentioned method. As a sub-analysis, we investigated the percentage of malignancy on FNAC and its association with MRI findings.

## Patients and methods

### Subjects

At the Cancer Institute Hospital, Japanese Foundation for Cancer Research, MRI is performed for diagnosis of the preoperative intraductal spread of breast cancer and for cases of abnormal nipple discharge in which lesions are not detected by MMG or US. From January 2013 to September 2015, breast MRI examinations were conducted on 3817 patients. For 292 lesions, the radiological interpretation specialists judged that a second-look US examination was necessary to determine treatment. We investigated 235 of those lesions for which consent to perform second-look US examination was obtained from the attending physician and the patient. The lesions comprised 151 preoperative ipsilateral breast lesions, 60 preoperative contralateral breast lesions, and 24 lesions with bloody nipple discharge. The mean patient age was 49.7 years (range 21–83).

## Methods

### MRI and interpretation

MRI was performed using two 3.0 T scanners (Canon Vantage Titan, GE Healthcare Discovery MR 750W) and one 1.5 T scanner (GE Healthcare Sigma HD). Breast coils were used with the subject in the prone position. The detailed imaging conditions are shown in Table 1. MRI interpretation was performed by two radiological interpretation specialists according to the criteria established in Breast Imaging-Reporting and Data System (5th edition). We targeted lesions that were newly detected by MRI and required evaluation of benign or malignant status. The following imaging procedures were performed.

**Table 1:** The detailed imaging conditions of MRI

Model	Method	Cross-section	Sequence	Fat suppression	TR	TE	FA	FOV	Matrix	Slice thickness	Gap
Canon Vantage Titan (3.0 T)	T2WI	Coronal	2DFSE	SPAIR	8801	75	90	250/340	352/240	3.0	0
	T1WI	Coronal	3DFFE		6.5	2.7	12	250/340	348/240	3.0	− 1.5
	T1WI dynamic	Coronal	3DFFE	Enhanced fat free	5.9	2.7	13	250/340	216/256	1.5	− 0.75
	High resolution	Axial	3DFFE	Enhanced fat free	6.2	3.2	12	200/340	424/240	2.0	− 1.0
GE Discovery MR 750w (3.0 T)	T2WI	Coronal	IDEALwater		6672	83	111	340	352/224	3.0	0
	T1WI	Coronal	FSPGR		4.9	2.1		340	416/320	3.0	− 1.5
	T1WI dynamic	Coronal	FSPGR	SPECIAL	5.4	1.7	20	340	352/224	1.6	− 0.8
	High resolution	Axial	VIBRANT FLEX	2-point Dixon	6.8	1.8		340	300/420	2.0	− 1.0
GE Signa HD (1.5 T)	T2WI	Coronal	2DFSE	CHESS	3600	85	90	260	384/224	3.0	0
	T1WI dynamic	Coronal	Gradient echo	SPECIAL	3.6	1.0	15	260	320/224	4.0	− 2.0
	High resolution	Axial	VIBRANT	SPECIAL	4.9	2.3	10	350	352/320	2.0	− 1.0

*FSE* fast spin echo, *FSE* fast spin echo, *IDEAL* iterative decomposition of water/fat using echo asymmetry and least squares estimation, *FSPGR* fast spoiled GLASS, *VIBRANT* volume imaging for breast assessment, *SPAIR* spectral attenuated with inversion recovery, *CHESS* chemical shift selective

### Confirmation of targets and indicators

Targets were confirmed based on MRI findings. The indicators used in the present study to identify the targets were classified as follows:

- Classical landmarks (for targets confirmed on subtraction maximum intensity projection images, contrast dynamic coronal-section images, and high-resolution images): the target shape, positional relationship between the target and the main lesion, and the nipple. Cysts, dilated ducts, and fibroadenomas surrounding targets were confirmed on T2-weighted images.
- Surrounding tissue landmarks (for targets confirmed on high-resolution images): the glandular pattern, adipose morphology, Cooper’s ligaments, and vascular routes as anatomical breast structures surrounding the target.

### Prediction of positional changes of targets and indicators on MRI and ultrasonographic images

The shape of the breast considerably changes with the body position, and the degree of change is not uniform. Therefore, to predict positional changes of the target and indicators based on MRI and US, it is essential to confirm the mammary gland distribution, lateral border line, and boundary surface between lobes.

**Fig. 1:**
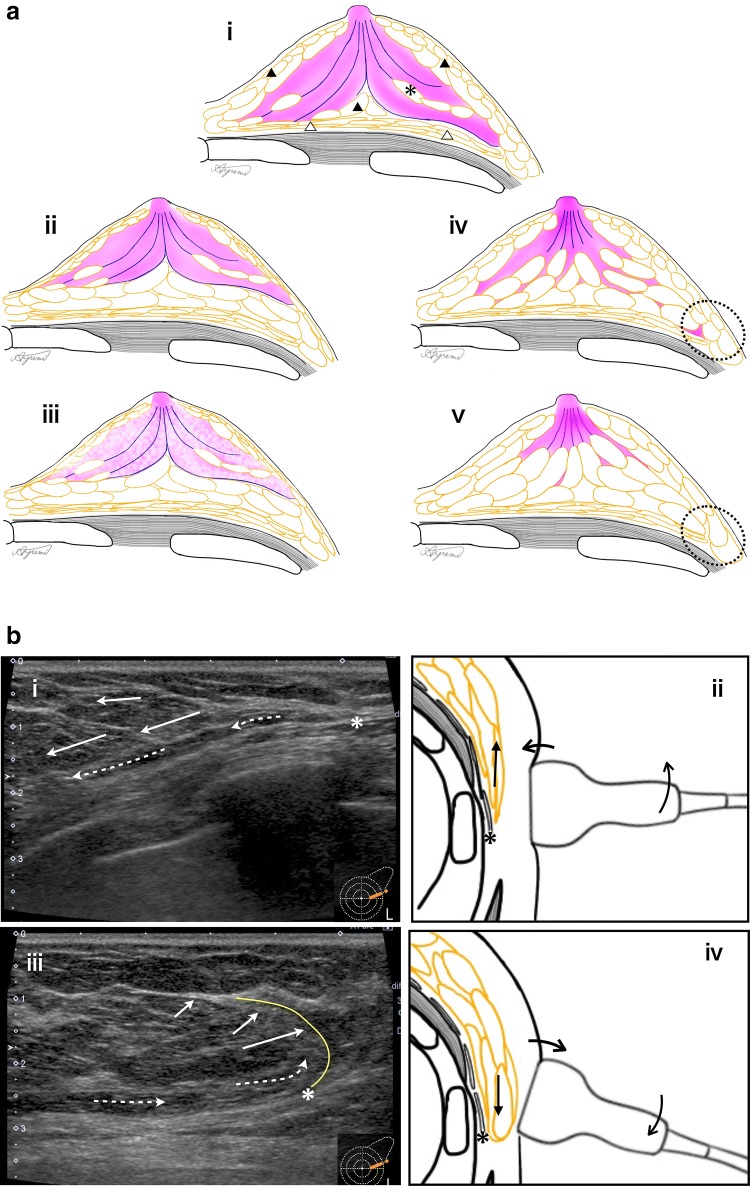

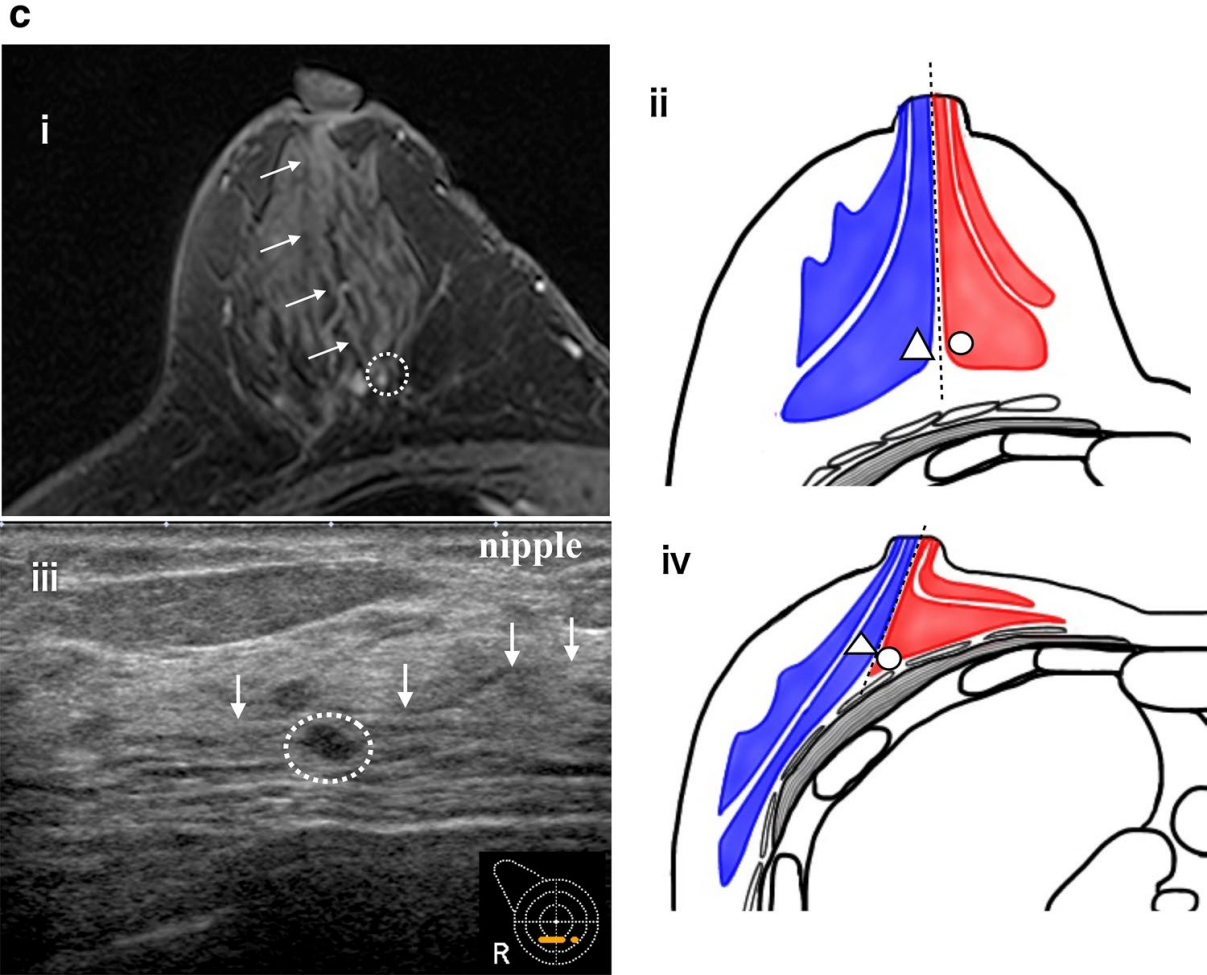
**a** Image for understanding fat and mammary gland distribution in breast image. i The fibroglandular zone is distributed to the periphery. The mammary lateral and posterior border line are clear. Some fatty lobules (asterisk) are partially visible between the glandular lobes. *PAFS* protective adipofascial system (filled triangle), *LAFS* lubricant adipofascial system (open triangle) [14]. ii The mammary posterior fats are wealthy. The mammary lateral and posterior border line are clear. iii Fat is mixed in the fibroglandular zone. The mammary lateral and posterior border line are clear. iv The fibroglandular tissues are distributed between multiple fat lobules, and the lateral and posterior fat boundaries are ambiguous. v The fibroglandular tissues are mainly distributed near the nipple, and the fibroglandular tissues between the fat lobules are barely visible. Sometimes, mammary gland distribution patterns shown in (i–v) are mixed in one breast. Use the **b** technique to detect the fatty breast lateral border line (iv and v dotted circles) on ultrasound images. **b** Handling technique for finding the lateral border line of fatty breasts. i, ii With the probe pressed, the lateral border line is obscured because the dorsal fat and breast fat look similar in shape. iii, iv When the inner pressure of the probe is released, the adipose layer of the retro-mammary space, LAFS (dotted arrow), slides and only the fat in the breast (arrow) is displaced outward. Then, a curve (yellow line) from the outer boundary of LAFS (asterisk) appears. Repeat the handling of (ii) and (iv) several times. **c** In the supine position, the major pectoral muscle side of the breast, more so than the skin side, is displaced along the curve of the chest wall to the outside. i In MRI, the lesion is at the lobular interface under the nipple. ii Schematic diagram of (i). iii In ultrasound, the lesion is outwardly displaced from the nipple. The lobular interface is inclined outward from the nipple. iv Schematic diagram of (iii)

When the body is in the supine position, the major pectoral muscle side of the breast, more so than the skin side, is displaced along the curve of the chest wall to the outside. This is because the fixation of the adipose layer of the retro-mammary space and breast (LAFS: lubricant adipofascial system [14]) is extremely loose compared with the fixation of the breast and the skin by Cooper’s ligaments. Because the degree of displacement does not correlate with the composition and size of the breast, it is necessary to confirm the mammary gland distribution and lateral border line as a whole image (Fig. 1a, b).

In addition, the degrees of positional-change displacement of the front and back lobes of the breast differ, with the back lobe being more displaced. Confirming whether the target and/or indicators are in the front or back lobe of the breast is important for predicting their positional changes (Fig. 1c). Therefore, it is necessary to confirm the boundary surface between the front and back lobes (lobular interface). In MRI, the lobular interface can be confirmed from high-resolution transverse images and visualized as a continuous linear structure running from below the nipple to the outside. In US, at the time of sweep observation, the interface is detected as a boundary surface that differs from the “TDLU-duct-surrounding stroma pattern”; it is occasionally confirmed as a partial sheet-like hyperechoic image [15]. In cases where it is difficult to confirm the interface, it can be detected as a lateral deviation of approximately 1–3 mm by changing the angle of pressure applied with the probe. Because lateral deviation is easier to recognize in breast sites with more fibrous components and stroma matrix components, in most breasts, it is easier to identify the lobular interface beneath the nipple and in the upper lateral area.

### Identification of targets by second-look US

A Canon Ultrasound Diagnostic System Aplio500 (Tochigi, Japan) attached to a probe (PLT-1204BX; center frequency 14 MHz) was used for all US examinations. Examinations were performed by clinical laboratory technicians under the direction of a specialist in breast US (A.I.). Specific targets were identified via the following procedures.

First, we used classical landmarks to conduct a US search for targets.

**Fig. 2:**
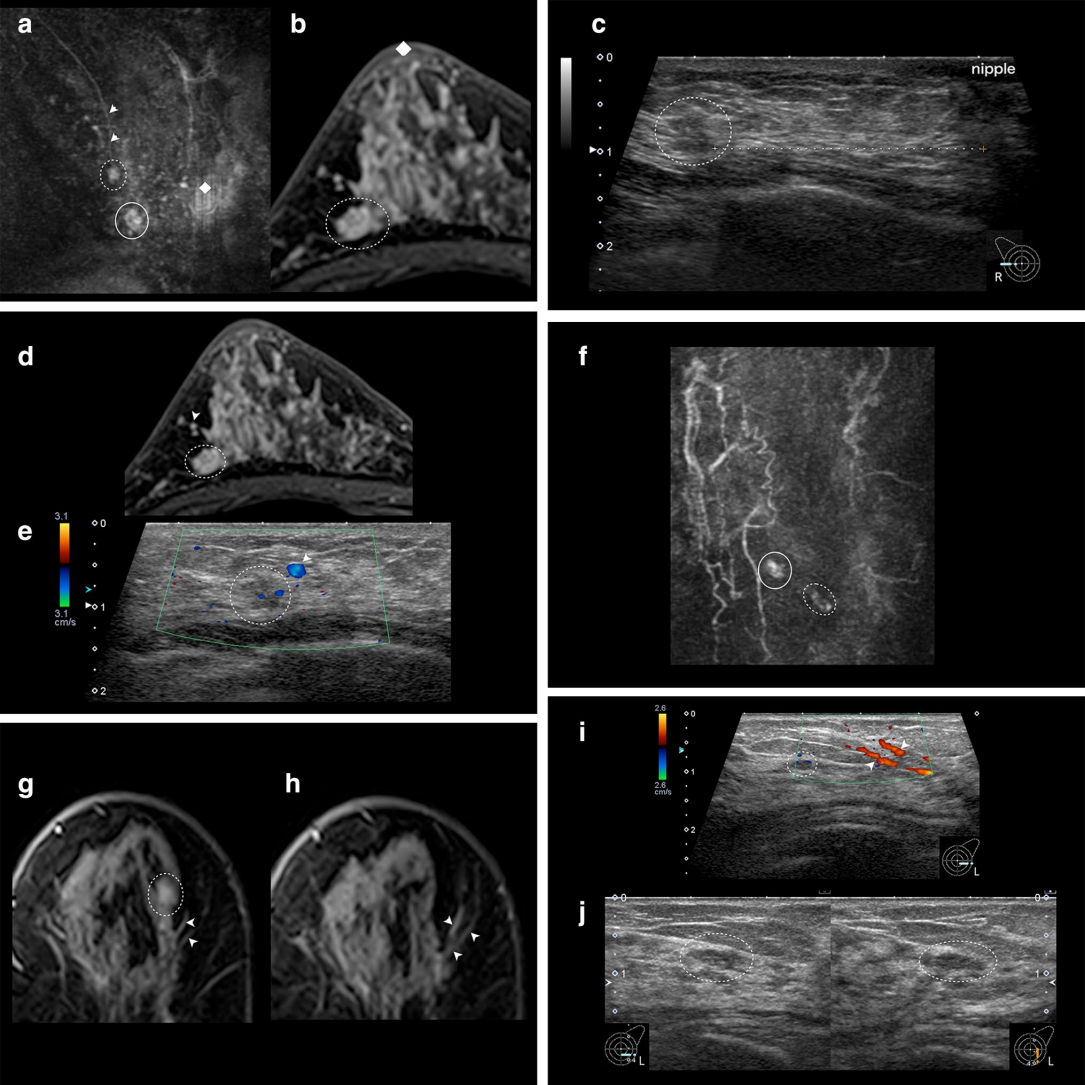
**a** The subject is a 48-year-old Japanese woman. Right breast. MRI subtraction MIP. Main lesion (round line), MRI-detected lesion: target (dotted circles), nipple (filled diamond), blood vessel from axilla (arrow). Background BPE is marked. **b** Search for classical landmarks on high-resolution MRI. As landmarks, the positional relationship from the nipple (nipple) and the shape of the target (findings) should be in contact with the anterior fat and posterior fat. **c** US identified the target (dotted circles) using the positional relationship with the nipple and characteristic findings as indicators. **d**, **e** The target (dotted circles) was confirmed on the basis of its correlation with new anatomical landmark, i.e., the blood vessels (arrowheads in **a**, **d**, and **e**) running from the axilla, and its relationship with the surrounding adipose morphology. The lateral thoracic artery route from the axilla is clearly visible on both MRI and US. Therefore, it is easy to use as a landmark. FNAC: malignant; histological diagnosis: DCIS. **f** The subject is a 78-year-old Japanese woman. Left breast. MRI subtraction MIP. Main lesion is just under the nipple (round line), MRI-detected lesion: target (dotted circles). **g**, **h** Because the classical landmark was unable to detect the lesion image in US, a high-resolution MRI is searched for the surrounding tissue landmarks. Two characteristic blood vessels (arrows) merge in a V shape near the target. The target (dotted circles) is slightly cephalad from the site of the two merging vessels and is located on the surface side of the breast. **i** US confirms the two vessels merging in a V shape. The target is confirmed on the surface side of the breast cephalad to the point of merger and identified as the target because there are no other candidates. **j** The identified target is detected as an isoechoic, 5.8 × 6.2 × 2.2 mm oval. US findings indicate a benign lesion/mastopathy. FNAC: malignant; histological diagnosis: DCIS

When a target was detected, we observed the relationship with the other uninvestigated classical and surrounding tissue landmarks and identified the target in cases where the relationship was in agreement (Fig. 2a–e). In the absence of agreement, the target was not identified, and we proceeded to the next step.

When no target was detected or the relationship with other landmarks was not in agreement, identification was performed using the surrounding tissue landmarks. When this procedure identified a target candidate, we confirmed that there were no other candidate lesions. Thereafter, the size and shape of the lesion and the positional relationship between the nipple and main lesion were confirmed. If there was no disagreement, the target was identified (Fig. 2f–j).

### Cytological examination

When a target was identified, aspiration cytology was performed under US guidance using a 22G catheter needle for puncture. Cytological results were classified according to the 2003 Japanese Breast Cancer Society guidelines. First, the results were classified as “adequate” or “inadequate.” Thereafter, adequate results were further subclassified as normal or benign, indeterminate, suspicious for malignancy, or malignant.

### Decision on therapeutic approach

At the Breast Oncology Center conference, the treatment approach—excision biopsy, follow-up observation, or needle biopsy—was determined based on the cytological results, MRI findings, US findings, and positional relationship with the main lesion. Follow-up observation cases were advised to undergo a US exam every 6 months.

### Histopathological examination

Resected specimens were prepared as slices at intervals of 5 mm and examined histopathologically. The results were classified as malignant (IDC: invasive ductal carcinoma, DCIS: ductal carcinoma in situ) or not malignant (benign, normal) and then analyzed.

### Study items and statistical tests

The present study investigated relationships between the following variables: MRI findings and the target size, target identification rate, and the main US indicators that led to identifying the target; FNAC results and MRI findings; MRI findings and histopathological results; FNAC results and histopathological results; and FNAC results. For statistical analysis, the main US indicators that triggered the identification of the target were assessed using Student’s *t* test and other items were assessed using the Chi squared test. The level of statistical significance was set at *p* < 0.05.

This study was approved by the Ethics Committee of the Japanese Foundation for Cancer Research, and all patients provided informed consent.

## Results

### MRI findings and target size

Of the 235 total targets, the MRI findings indicated 93 focal (39.6%), 76 mass (32.3%), and 66 non-mass (28.1%) lesions. The mean size of the total targets was 9.3 mm. The focal, mass, and non-mass lesions had mean sizes of 4.2 mm (range 3–5 mm), 8.0 mm (5–33 mm), and 17.8 (3–82) mm, respectively (Table 2).

**Table 2:** Size, FNAC and histology by MRI findings

MRI findings^a^		Size (mm) (*n* = 235)	FNAC (*n* = 232)					Histology (*n* = 99)			
			Malignant (%)	Suspicious for malignancy (%)	Indeterminate (%)	Benign (%)	Inadequate (%)	Cancer (%)	(IDC)^b^	(DCIS)^c^	No malignancy
Total	235	9.3	55	3	18	149	7	70	19	51	149
Focus	93	4.2	15 (16.1)	0 (0)	6 (6.5)	70 (76.1)	1 (1.1)	16 (19.0)	4	12	68 (79)
Mass	76	8.0	24 (31.5)	3 (3.9)	4 (5.4)	39 (52.7)	4 (5.3)	31 (43.1)	12	19	41 (56.9)
Non-mass	66	17.8	16 (24.2)	0 (0)	7 (10.6)	41 (62.1)	2 (3.0)	23 (36.5)	3	20	40 (63.5)
			*p* = 0.101			*p* < 0.05		*p* = 0.089			*p* < 0.05

^a^BI-RADS 5th edition

^b^Invasive ductal carcinoma

^c^Ductal carcinoma in situ

### Target identification rate and main US indicators leading to the identification of targets

**Fig. 3:**
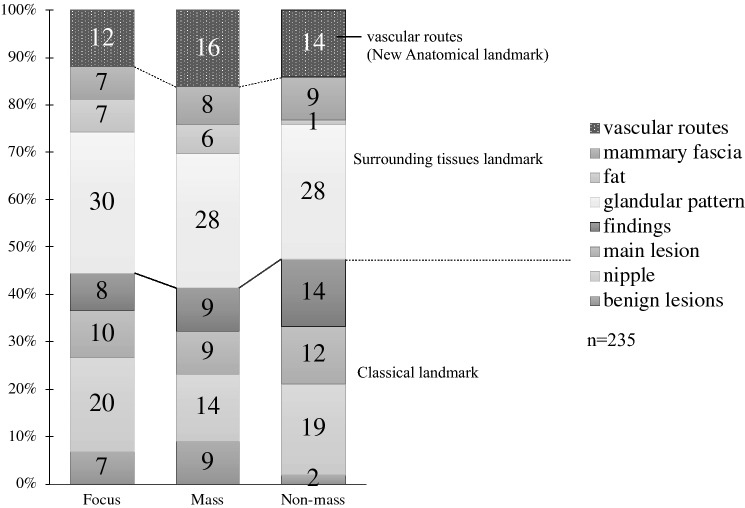
The main indicators that led to the identification of targets by US. The main US indicators showed the same trend regardless of the MRI findings. A glandular pattern was the most common landmark, accounting for 28–30% of the lesions identified. New anatomical landmarks, i.e., vascular routes, led to 12–16% of the lesions identified

The target was identified for 233 of 235 examined lesions (identification rate 99%). The main US landmarks that led to identification showed the same trends regardless of MRI findings (focal, mass, or non-mass lesions) (Fig. 3). A glandular pattern was the most common landmark, accounting for 28–30% of the lesions identified. Of the surrounding tissue landmarks, Cooper’s ligaments and adipose morphology led to the identification of 10–14% of the targets and new anatomical landmarks, i.e., vascular routes, led to the identification of 12–16% of the targets. The trends were similar for MRI findings. Approximately, 41–47% of the targets were identified based on conventionally used landmarks.

### Relationship between FNAC results and MRI findings

US-guided FNAC was performed for 232 lesions. One lesion was diagnosed as a hemangioma based on US findings. The FNAC results indicated 55 malignant lesions (23.7%), 3 suspicious for malignancy lesions (1.3%), 18 indeterminate lesions (7.6%), 149 normal or benign lesions (64.2%), and 7 inadequate (3.0%) lesions. On stratification based on the MRI findings, the malignancy rates by FNAC for focal, mass, and non-mass lesions were 16.1%, 31.5%, and 24.2%, respectively. Therefore, mass lesions showed a greater tendency to be malignant (*p* = 0.101), whereas focal lesions were significantly more likely to be benign (*p* < 0.05; Table 2).

### Therapeutic approach results and reasons for excision of targets

The therapeutic approaches undertaken for the lesions, stratified based on the cytological results by FNAC, were as follows: all 55 malignant lesions were excised; all 3 suspicious for malignancy lesions were excised; of the 18 indeterminate lesions, 10 were excised, 6 underwent CNB, and 2 were observed; of the 149 benign lesions, 36 were excised (8 lesions suspicious for malignancy on imaging, 11 lesions proximal to the main lesion, and 17 lesions excised at the time of mastectomy), 3 underwent CNB, and 110 were observed; and of the 7 inadequate lesions, 2 were excised (both at the time of mastectomy) and 5 were observed.

In the present study, nine lesions underwent CNB after FNAC. The histopathological results showed three indeterminate lesions and six benign lesions. Excision biopsy was performed on the three indeterminate lesions, of which the final pathological diagnosis was benign for two lesions and malignant for one lesion. The malignant lesion had previously been evaluated as indeterminate by FNAC.

### MRI findings and histopathological results

Overall, 13 lesions were excluded from the analysis of the histopathological results because total mastectomy was performed, therefore, histopathological diagnosis of the target could not be performed.

The histopathological results were stratified by MRI findings. The percentages of malignant focal, mass, and non-mass lesions were 19.0%, 43.1%, and 36.5%, respectively. Mass lesions showed a trend of a higher rate of malignancy (*p* = 0.089). Conversely, 79% of focal lesions were benign, which was significant (*p* < 0.05) (Table 2).

### Follow-up of the observed lesions

Follow-up observation was possible for 115 (95.0%) lesions. The mean follow-up period was approximately 2 years. During that period, none of the patients developed new lesions or showed changes in US findings. Accordingly, these lesions were judged to have been benign.

### FNAC results

Of the 51 lesions diagnosed as malignant by FNAC, 48 (94.1%) were also diagnosed as malignant by histopathological findings. Of the other lesions diagnosed by FNAC, 3/3 (100%) of lesions suspicious for malignancy, 9/18 (50%) of indeterminate lesions, 10/141 (7.1%) normal or benign lesions, and 0/6 (0%) of inadequate lesions were diagnosed as malignant based on histopathological findings (Table 3). The results for the cytological categories were as follows: inadequate rate, 2.7%; indeterminate rate, 8.5%; positive predictive value of suspicious of malignancy cells, 100.0%; sensitivity, 85.7%; specificity, 91.6%; negative predictive value of normal/benign cells, 92.9%; positive predictive value of malignant cells, 94.1%; false-negative rate, 14.3%; and false-positive rate, 2.1%. The overall accuracy rate was 89.7% (Table 4).

**Table 3:** FNAC results

Cytological category	Histology		
		Malignancy	No malignancy
Malignant	51	48	3
Suspicious for malignancy	3	3	0
Indeterminate	18	9	9
Normal or benign	141	10	131
Inadequate	6	0	6
Total	219	70	149

Thirteen lesions were excluded from the analysis of the histopathological results because total mastectomy was performed, and histopathological diagnosis for the target could not be performed

**Table 4:** The results for the cytological categories

		WG JSCC
Inadequate rate	2.7	17.7
Indeterminate rate	8.5	7.8
Positive predictive value of ‘malignancy suspected’ cells	100.0	92.4
Sensitivity	85.7	96.7
Specificity	91.6	84.3
Negative predictive value of ‘normal/benign’ cells	92.9	98.2
Positive predictive value of ‘malignant’ cells	94.1	99.5
False-negative value	14.3	3.31
False-positive value	2.1	0.25
Accuracy	89.7	88.0

Sensitivity = percentage of cases cytologically rated as ‘indeterminate’, ‘malignancy suspected’ or ‘malignant’ among all cases of ‘adequate’ samples histologically rated as ‘malignant’

Specificity = percentage of cases cytologically rated as ‘normal or benign’ among all cases of ‘adequate’ samples histologically rated as ‘non-malignant’

False-negative value = percentage of cytologically negative cases among all cases of ‘adequate’ samples histologically rated as ‘malignant’

False-positive value = percentage of cytologically positive cases among all cases of ‘adequate’ samples histologically rated as ‘non-malignant’

Accuracy = percentage of cases cytologically rated as ‘normal or benign’ and confirmed as benign by histology and cases cytologically rated as ‘indeterminate’, ‘malignancy suspected’ or ‘malignant’ and confirmed as malignant by histology among all cases of ‘adequate’ samples histologically rated as ‘non-malignant’ and ‘malignant’

*WG JSCC* The Working Group of the Japanese Society of Clinical Cytology. Rin Yamaguchi

## Discussion

Lesions identified by MRI are often small in size and unclear. Moreover, because body position differs between examinations, it can be difficult to accurately match lesions depicted by MRI with second-look US findings.

In reports where second-look US was performed with reference to the location, shape, and size of MRI-determined lesions, the identification rates are typically quite low at 23–71% [9–13]. Candelaria et al. have performed second-look US with reference to MRI findings using the distance of the lesion from the nipple and its depth. However, because these values are greatly affected by posture, the identification rate was only 67% [16]. However, in a Korean study, Hong et al. have reported a high identification rate of 86.8% [17]. The authors suggested that their superior identification rate is attributable to the better suitability of smaller breasted Asian women for US exams or recent advances in US devices. In addition to the conventional identification method, Hong et al. have noted that they used subcutaneous fat, glandular tissue, and subglandular fat as breast landmarks in second-look US, which could have contributed to their superior identification rate.

In the present study, we added the positional relationship of targets with the surrounding vascular routes as a new landmark and achieved an identification rate of 99% in Asian women. This identification rate is higher than that reported by Hong et al. [17] for a similar population. Of note, approximately 15% of breast lesions were identified using vascular routes as landmarks, regardless of MRI findings. The use of vascular routes enabled the identification of lesions even in breasts where the characteristic adipose tissue, mammary gland distribution, and Cooper’s ligaments could not be detected. Therefore, this method contributed to our extremely high identification rate.

In the published literature, the mean size of lesions on MRI was 8.5–10.1 mm for masses and 22–32.6 mm for non-masses [9, 16–19]. In our study, the mean sizes were 8.0 mm for mass lesions and 17.8 mm for non-mass lesions, indicating that we were able to detect smaller lesions. Furthermore, we achieved an extremely high identification rate even for focal lesions < 5 mm. In previous reports, when stratifying lesions based on MRI findings, mass lesions often have a higher identification rate than non-mass lesions [9, 10, 12, 16, 17, 19, 20]. In our study, the identification rate was high irrespective of MRI findings, indicating that using the surrounding anatomical structures as indicators can enable reliable identification of even slightly pale lesions that do not exhibit malignant properties on US. In summary, these results highlight the importance of considering anatomical breast structures, including vascular routes, surrounding the target when performing second-look US examinations for MRI-detected breast abnormalities.

The method used for pathological diagnosis is also important. In reports to date, lesions found by second-look US have been diagnosed by CNB, VAB, and even open biopsy [5, 9, 11, 16].

We performed FNAC diagnosis of all lesions identified as targets in this study. Thereafter, we compared our FNAC results with those generated by the analysis of 10,890 cases at 12 facilities compiled by the Japan Society of Clinical Cytology Working Group (WGJSCC) [21].

It is impossible to compare the sensitivity in detail, because the WGJSCC did not study lesions with a US diameter ≤ 1 cm. Regarding false negatives, the WGJSCC study reported a rate of 15.5% (46/297) for tumors or hypoechoic lesions with a US diameter ≤ 1 cm. Our false-negative rate of 15.4% (8/52) was similar for lesions with a US diameter ≤ 1 cm (5.6 ± 2.0 mm). All ten false-negative cases in our study were low-malignancy DCIS, and there were no invasive carcinoma false negatives. Moreover, we reviewed three false-positive cases. The histopathological results showed mastopathy-type fibroadenoma, mastopathy accompanied by apocrine metaplasia, and various findings of mastopathy, consistent with the WGJSCC report [21].

In previous post-MRI second-look US studies, 70–80% of the lesions examined were found to be benign [8–10, 13, 16, 20, 22, 23] and in some cases, the targets were plural sites. FNAC is superior to CNB for puncture of lesions proximal to the skin and nipple, lesions at thin mammary gland sites (particularly on the internal side in small-breasted East Asian women), lesions proximal to thick blood vessels, and lesions in breasts with high-density fibrous components in MMG. In addition, FNAC is safe for patients undergoing oral anticoagulant therapy and for puncturing most lesions. Furthermore, FNAC allows faster diagnosis and is an economical inspection method.

Taken together, our findings support that FNAC is useful as an initial pathological diagnostic tool, even in cases of post-MRI second-look US. The finding that lesions difficult to diagnose by FNAC tend to be lobular carcinomas, sclerosing adenosis, low-malignancy DCIS, or mastopathy-type fibroadenomas should be considered [20]. Thus, by carefully considering the image findings, it is crucial to decide whether CNB or surgical resection is required or if the case should simply be observed.

Finally, the limitations of this study must be noted. First, this was a retrospective study and was thus biased in that all decisions, such as whether or not to perform second-look US, were made by the physician. Second, although excellent results were obtained when FNAC was combined with post-MRI second-look US using the new anatomical landmark as the indicator, enabling this technique at other institutions would not be simple because it is necessary to develop a uniform MRI protocol, standardize the image quality of US, and improve the series of techniques for FNAC.

Furthermore, we found that accurate diagnostic results could be obtained by FNAC, which is a minimally invasive technique. In the future, a multicenter reproducibility test should be performed to confirm our findings.
